# Albuminuria in Variola

**Published:** 1871-12

**Authors:** 


ALBUMINURIA IN VARIOLA.—In the *Lyon Medical*, of September, M. Cartaz speaks as follows of the occurrence of albumen in the urine in small-pox. Albuminuria is met with its confluent variola, in one out of every five cases. It is transitory usually, and has no effect on the progress of the disease. When permanent it brings with it the disorders noticed in Bright's disease. Even when it is only a passiug affection it may give rise to grave accidents, such as eclampsia, etc. In hæmorrhagic variola, albuminuria is constantly noticed, whether it be due to a lesion of the kidneys, or to a mixture of blood with the urine, or to both causes combined. M. Perroud, of Lyons, says that albuminuria may appear in the eruptive period, or in the period of desquamation. In the first case it is probably due to reflex cause ; in the second, to variolic poison. As in syphilis, variola has its secondary period characterized by multiple outbreaks on the surface of the skin (cutaneous rashes, pustulæ, sore throat, conjunctivitis) its tertiary or visceral period (orchitis, nephritis, pneumonia), and its intermediate period (deep inflammations of the skin, abscess, and iritis). Lastly, like syphilis, variola may be inoculated, and then it is its primitive accident (pustule of inoculation) or it is developed by infection, and then, like hereditary syphilis, it has no primary accident. The lesion of the kidney is the cause of albuminuria in small-pox. Generally, this lesion is slight, and yields easily to local and general tonics, such as iron, tannin, and quinine. M. Soulier remarks that the hypothesis that the albuminuria is renal is preferable, and he has found it true to nature.' In an autopsy of variola with albuminuria, he had found a lesion like that in Bright's disease. If albuminuria were caused by blood-lesion, it would be much more common and more fatal in variola. If, on the contrary, it were the result of renal lesion, we can better comprehend that as this lesion does not take place in all cases, albuminuria does not always exist. He thinks the best treatment for it is rubbing with warm oil.—*The Doctor.*

